# Supplementary material for: Functionally antagonistic polyelectrolyte for electro-ionic soft actuator
Source: Nat Commun. 2024 Jan 10;15:435. doi: 10.1038/s41467-024-44719-z (PMC10781978; doi:10.1038/s41467-024-44719-z)
Supplement: Supplementary file 1 — Supplementary Information [file 41467_2024_44719_MOESM1_ESM.pdf]

Supplementary Information

Title

Functionally Antagonistic Polyelectrolyte for Electro-Ionic Soft Actuator

Authors

Van Hiep Nguyen[1], Saewoong Oh[1], Manmatha Mahato[1], Rassoul Tabassian[1,2], Hyunjoon Yoo[1], Seong-Gyu Lee[3], Mousumi Garai[1], Kwang Jin Kim[4] and Il-Kwon Oh[1,*]

Affiliations

[1] National Creative Research Initiative for Functionally Antagonistic Nano-Engineering, Department of Mechanical Engineering, Korea Advanced Institute of Science and Technology (KAIST), 291 Daehak-ro, Yuseong-gu, Daejeon 34141, Republic of Korea

[2] Department of Mechanical and Production Engineering, Aarhus University, Katrinebjergvej 89 G-F, 8200 Aarhus N, Denmark

[3] Transmission Electron Microscopy Laboratory, KAIST Analysis Center for Research Advancement, Korea Advanced Institute of Science and Technology (KAIST), 291 Daehak-ro, Yuseong-gu, Daejeon 34141, Republic of Korea

[4] Active Materials and Smart Living Laboratory, Department of Mechanical Engineering, University of Nevada, Las Vegas (UNLV), Las Vegas, NV, 89154, USA

Contact info

* Correspondence and requests for materials should be addressed to I.-K. Oh (Email: ikoh@kaist.ac.kr)

Supplementary Figures and Tables

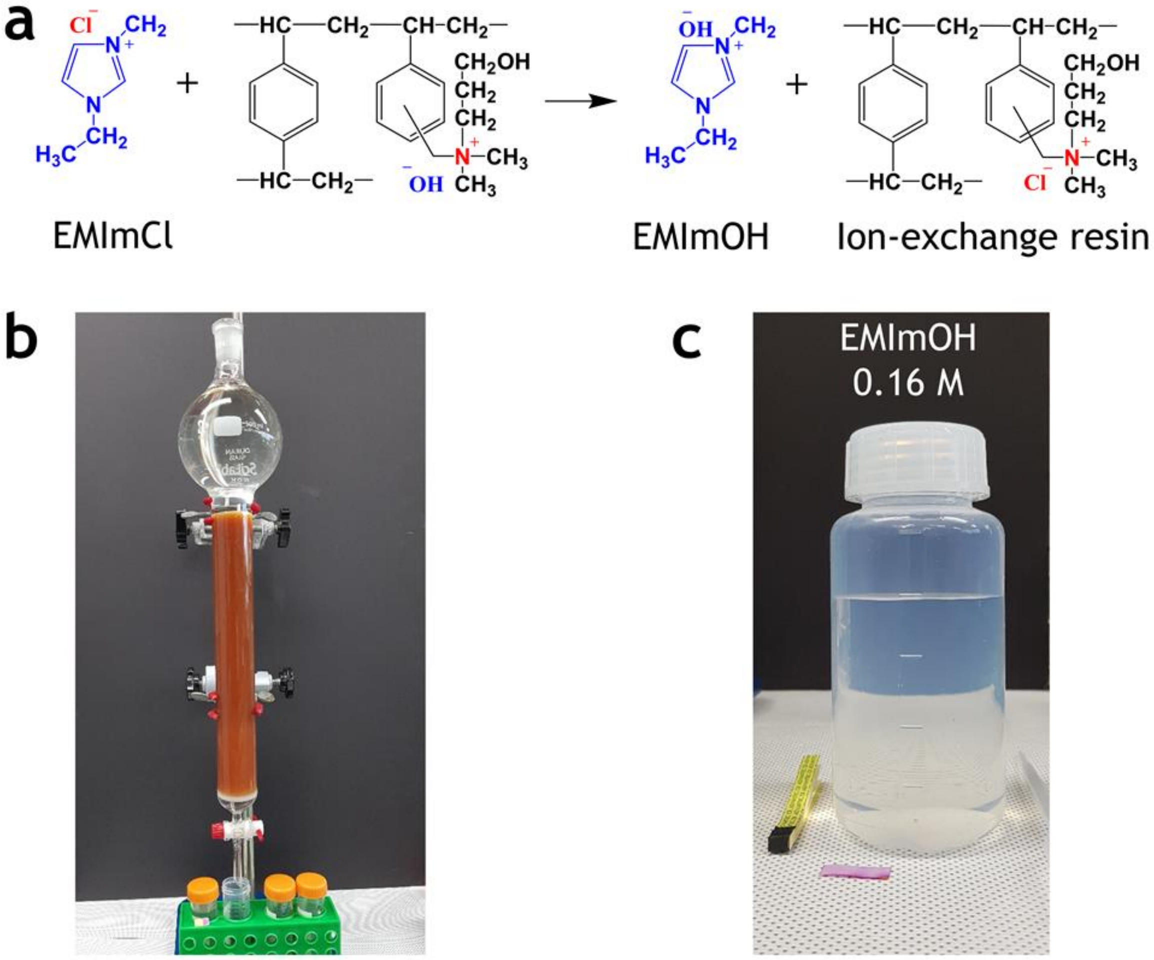

Supplementary Fig. 1. Preparing 1-ethyl-3-methylimidazolium hydroxide (EMImOH) by ion-exchange reaction. a. Scheme for ion-exchange reaction. **b.** Experimental setup for ion-exchange reaction. **c.** EMImOH product.

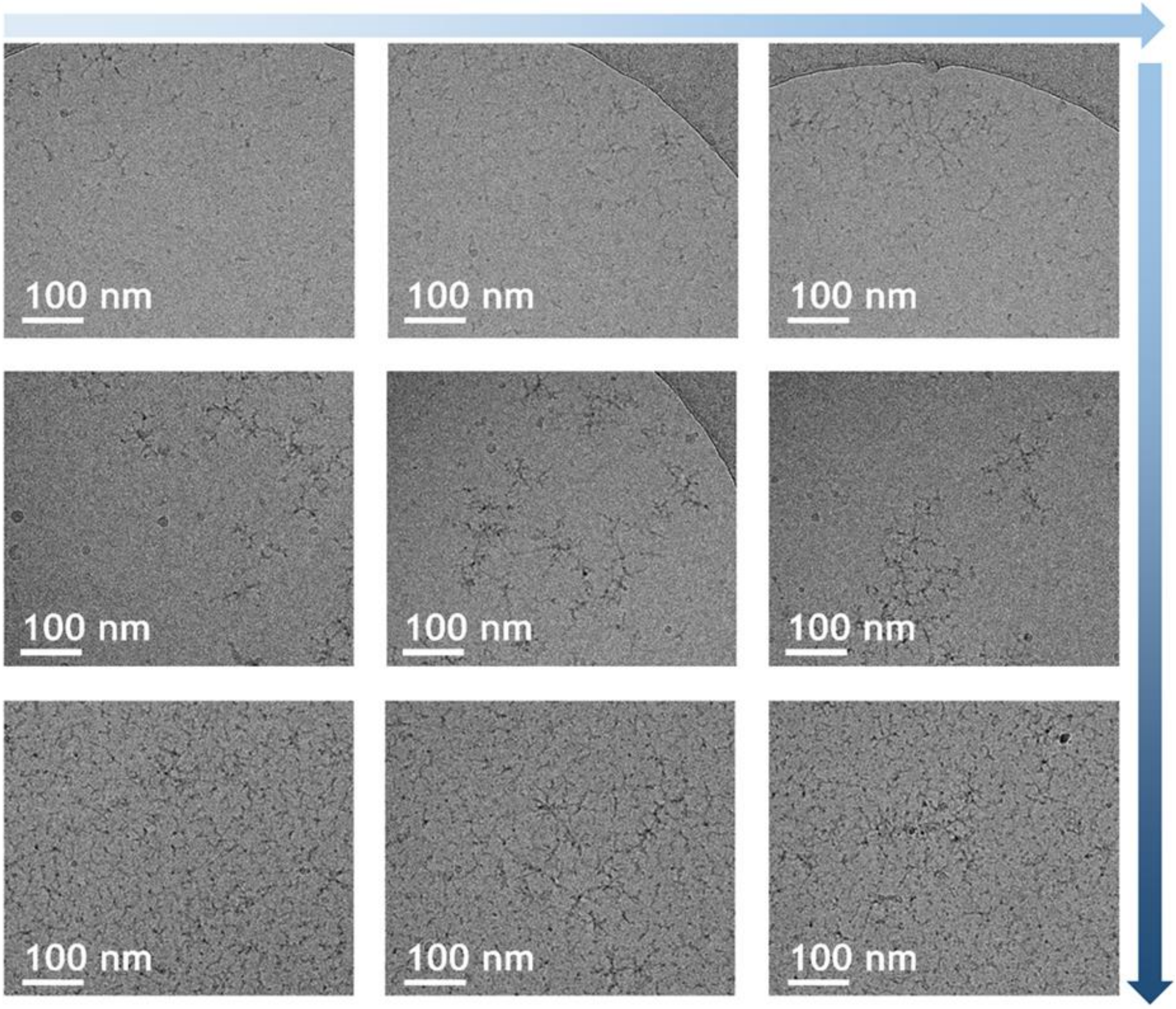

Supplementary Fig. 2. Cryo-TEM images of Nafion micelles at different concentrations. The arrow directions indicate the increases of concentration.

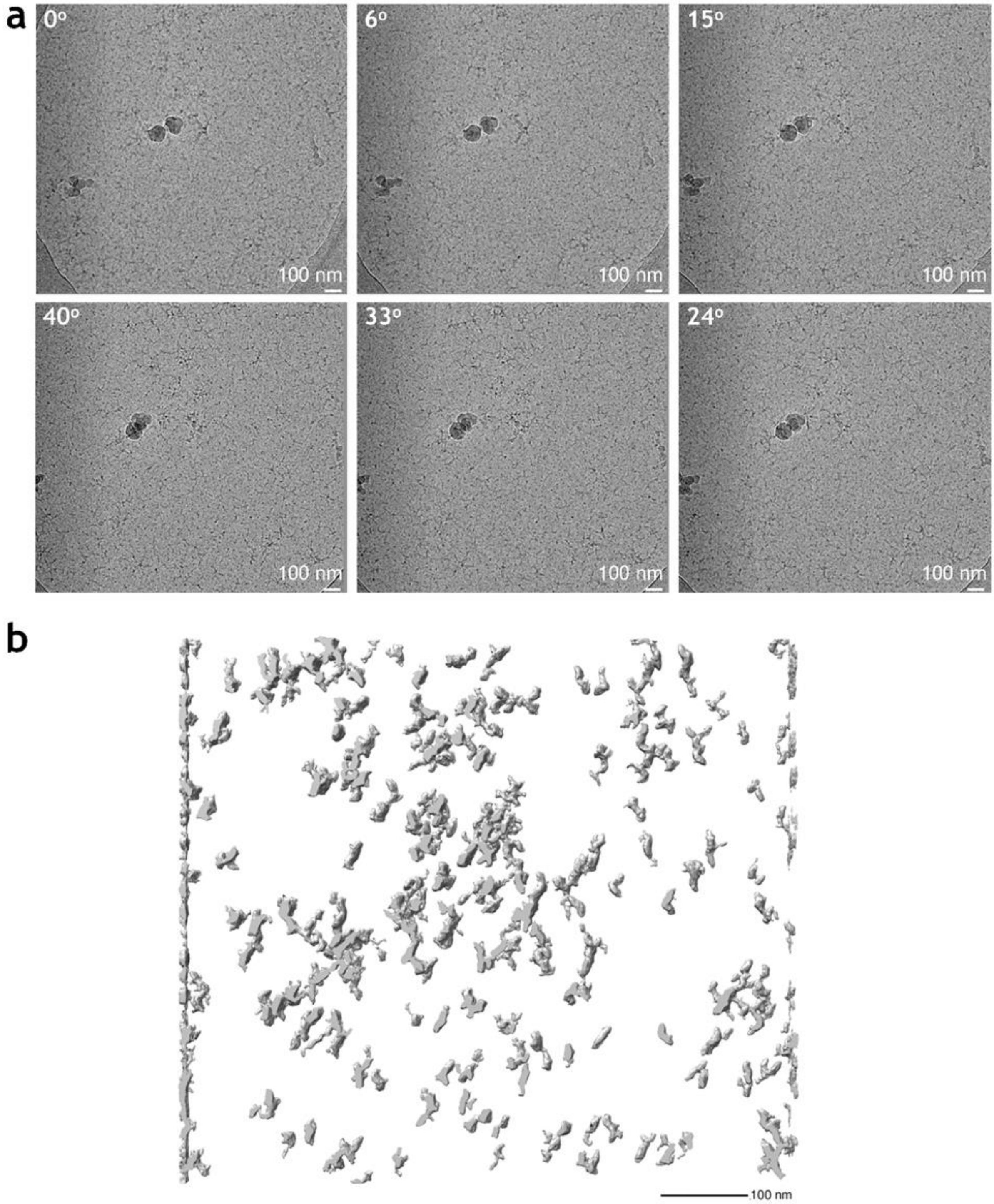

Supplementary Fig. 3. Cryo-TEM images of Nafion micelle. a. Images at the same position and different tilting angles. **b.** 3D image reconstructed from cryo-TEM tomography using software. The 3D image shows distorted micellar shapes in comparison to the real ones due to some noises while reconstructing from raw data with the help of software.

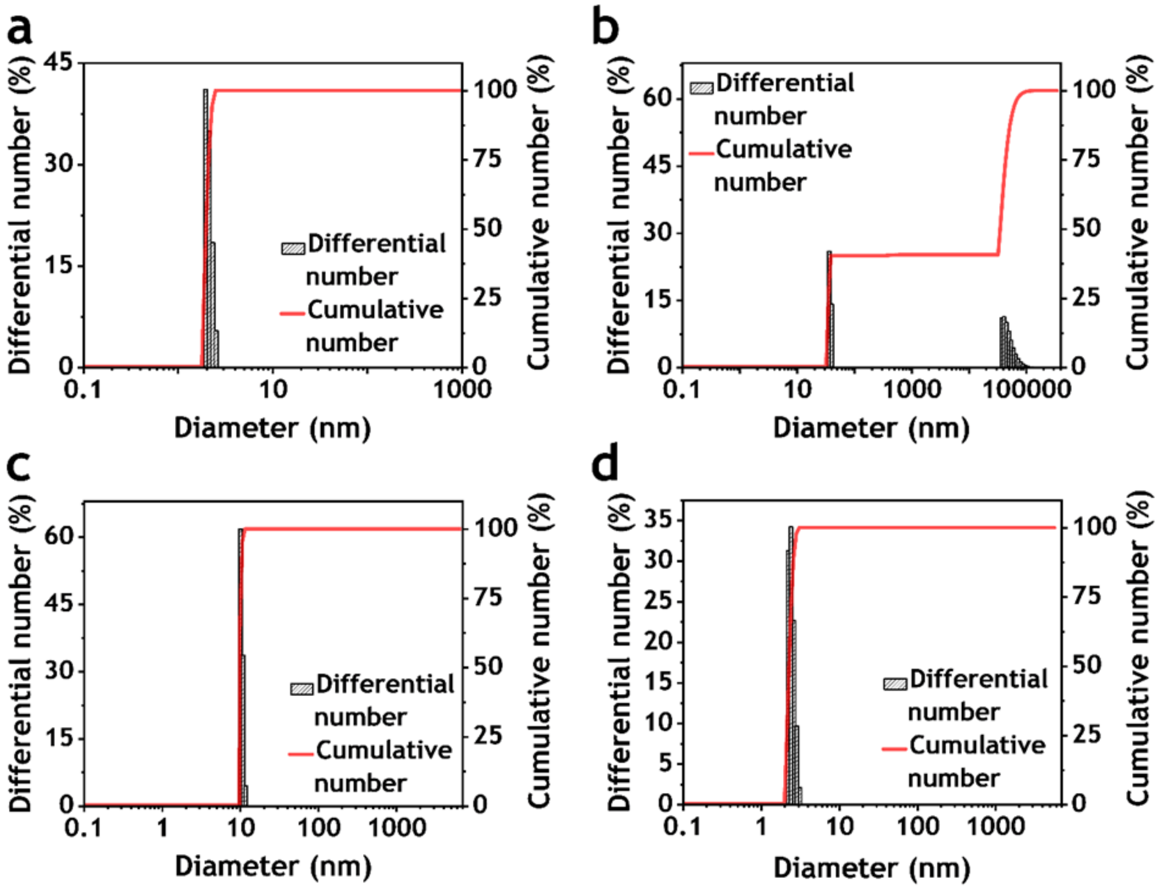

Supplementary Fig. 4. Particle size by dynamic light scattering (DLS). Nafion dispersions in water **a.** Before and **b.** After adding $EMImBF_4$ ionic liquid. Nafion dispersions in DMAc **c.** Before and **d.** After adding $EMImBF_4$ ionic liquid.

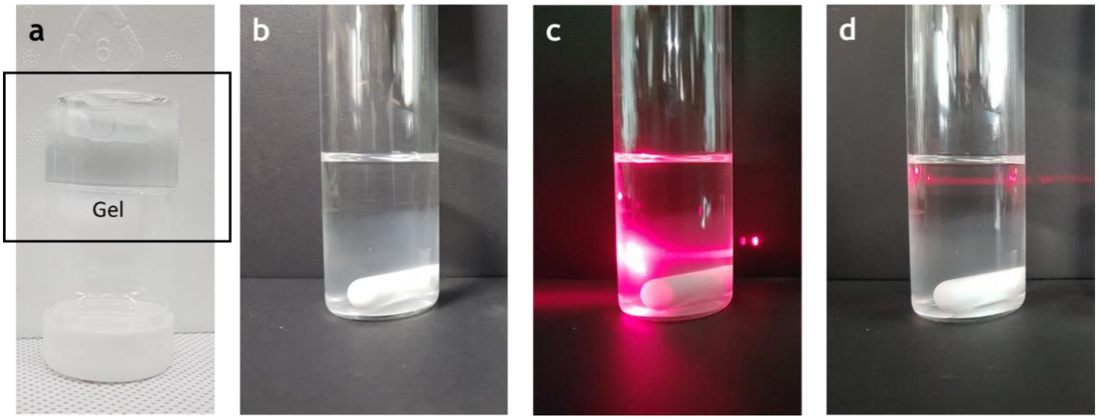

Supplementary Fig. 5. Effect of water content on micellar rheology. a. strong Nafion gel in low water content. **b**. precipitated Nafion micelles in high water content. **c**. Laser scattering of precipitated Nafion micelles in high water content. **d**. Laser propagation at high position.

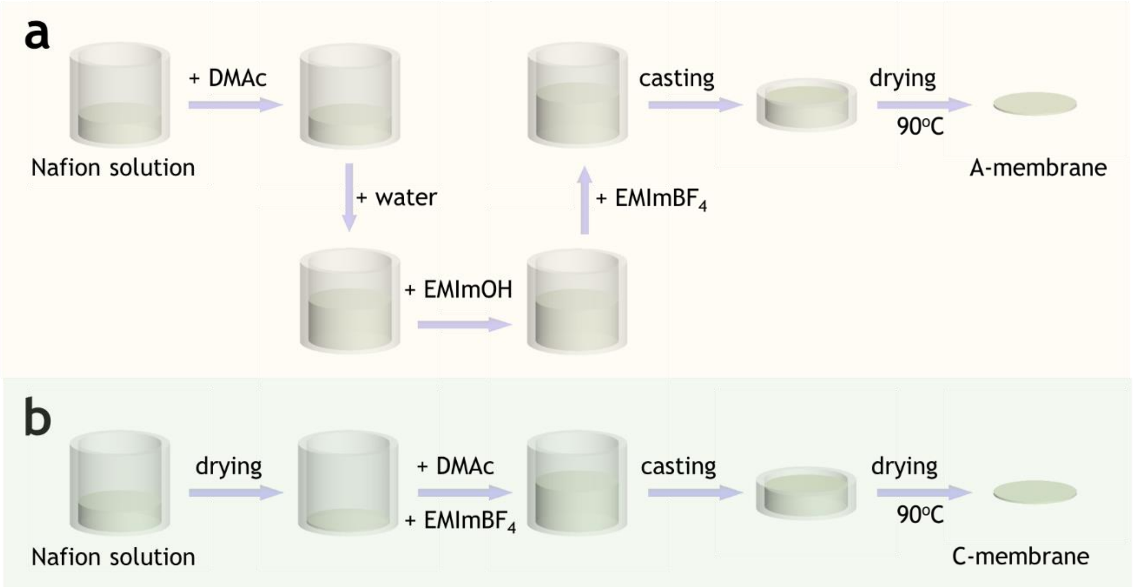

Supplementary Fig. 6. Procedures for fabricating polyelectrolyte membranes. a. Antagonistic membrane (A-membrane). **b.** Conventional membrane (C-membrane).

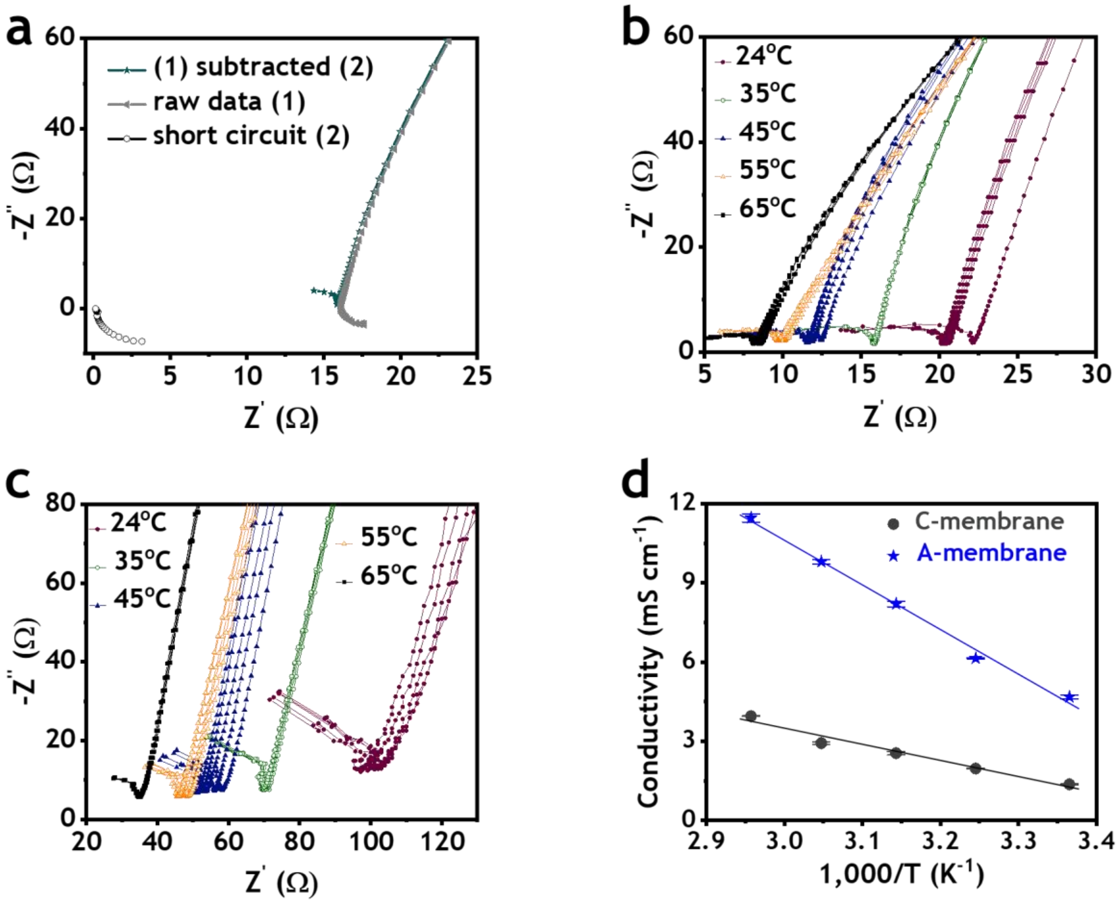

Supplementary Fig. 7. Ionic conductivity. **a.** Calibration of raw electrochemical impedance spectroscopy (EIS) data. **b.** EIS spectra of A-membrane. **c.** EIS spectra of C-membrane. **d.** Ionic conductivity calculated from EIS data.

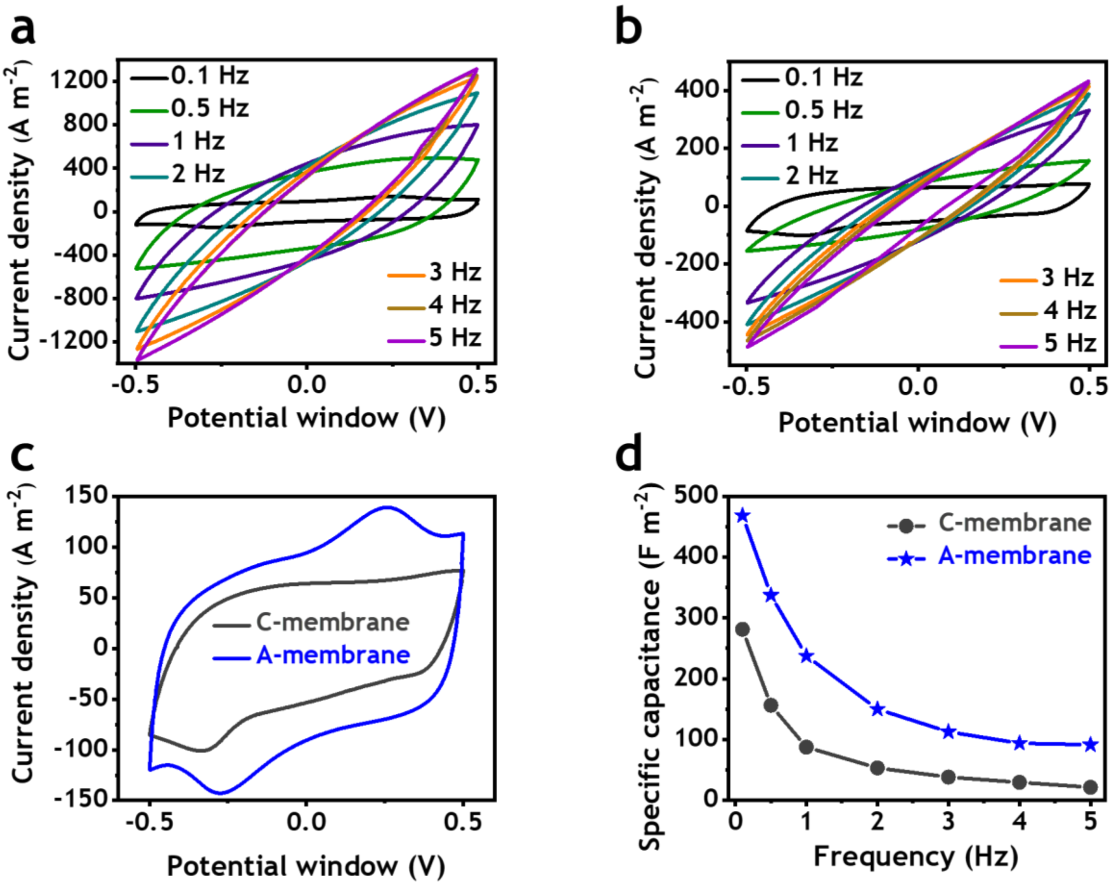

Supplementary Fig. 8. Specific capacitance of actuators. a. Cyclic voltammetry (CV) curves of A-membrane actuator. **b.** CV curves of C-membrane actuator. **c.** Typical comparison of CV curves of the two samples at 0.2 V s^{-1} (0.1 Hz)**. d.** Specific capacitance calculated from CV data.

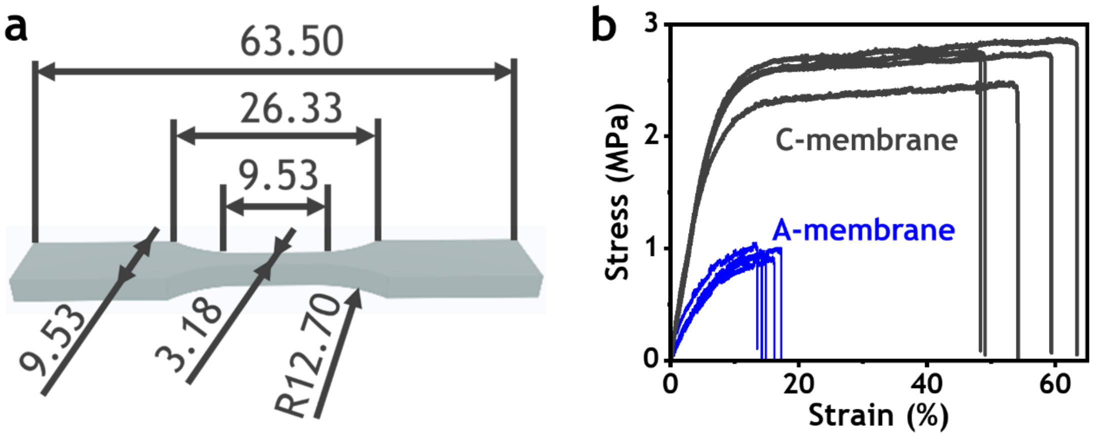

Supplementary Fig. 9. Mechanical properties. a. Dimensions of tensile test sample according to ASTM D638-V. **b.** Stress-strain curves.

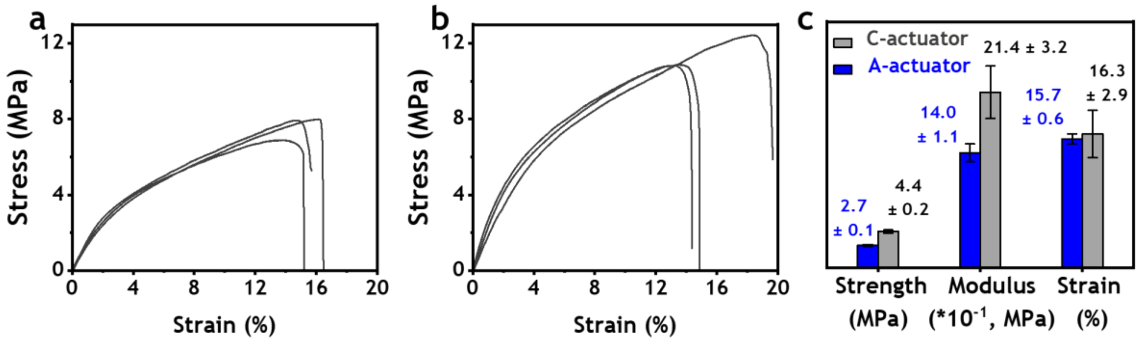

Supplementary Fig. 10. Mechanical properties of actuators. a. Stress-strain curves of A-actuator. **b.** Stress-strain curves of C-actuator. **c.** mechanical properties.

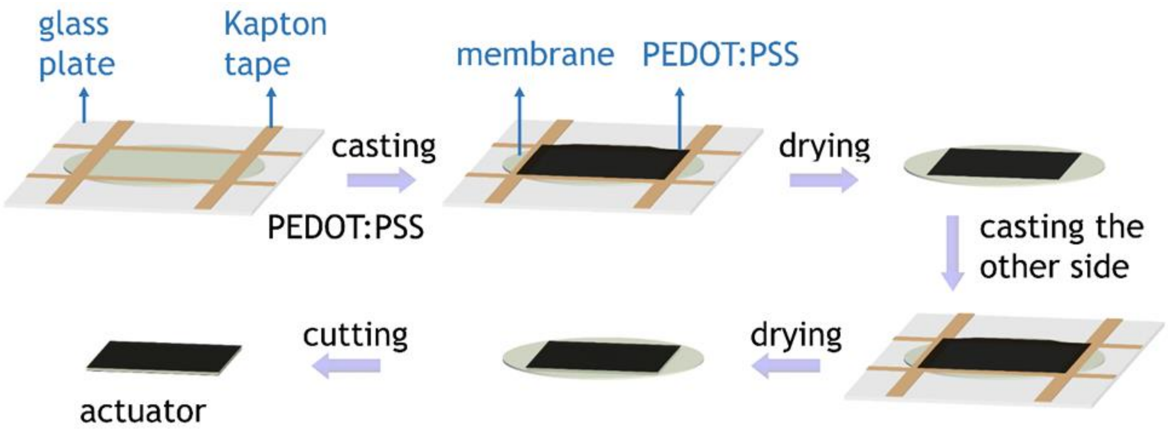

Supplementary Fig. 11. Procedure for fabricating actuator.

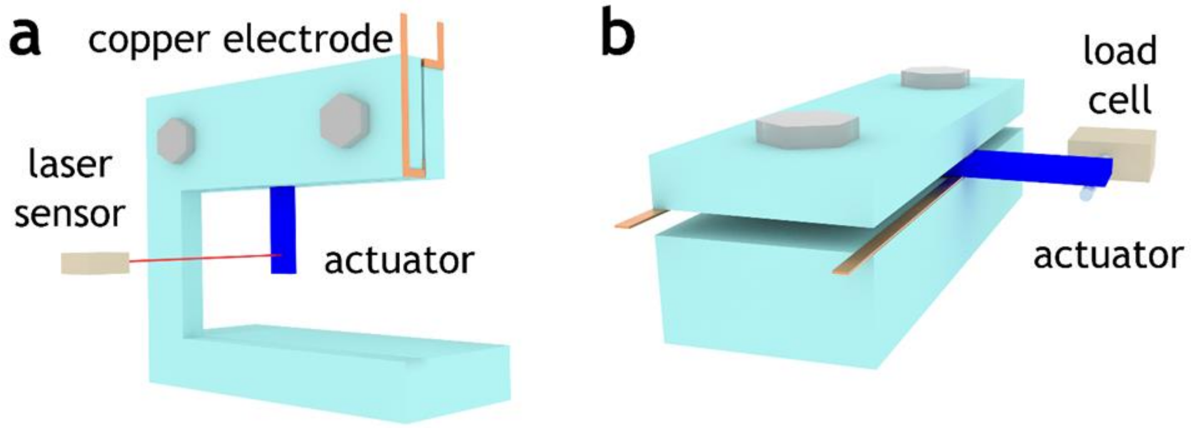

Supplementary Fig. 12. Actuator characterizations. Measurement setups for **a.** Bending displacement and **b.** Blocking force.

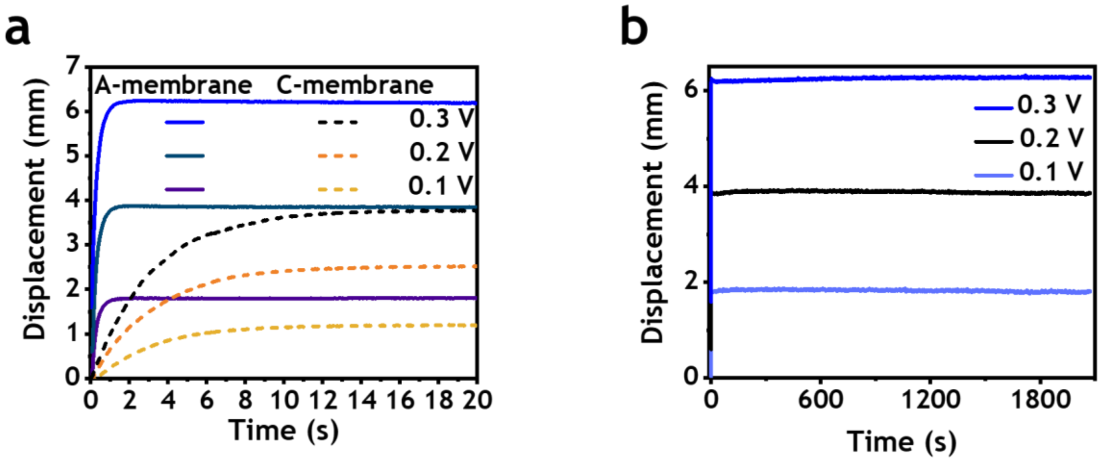

Supplementary Fig. 13. Direct current (DC) response. a. DC response at different voltages. **b.** DC response over 35 minutes.

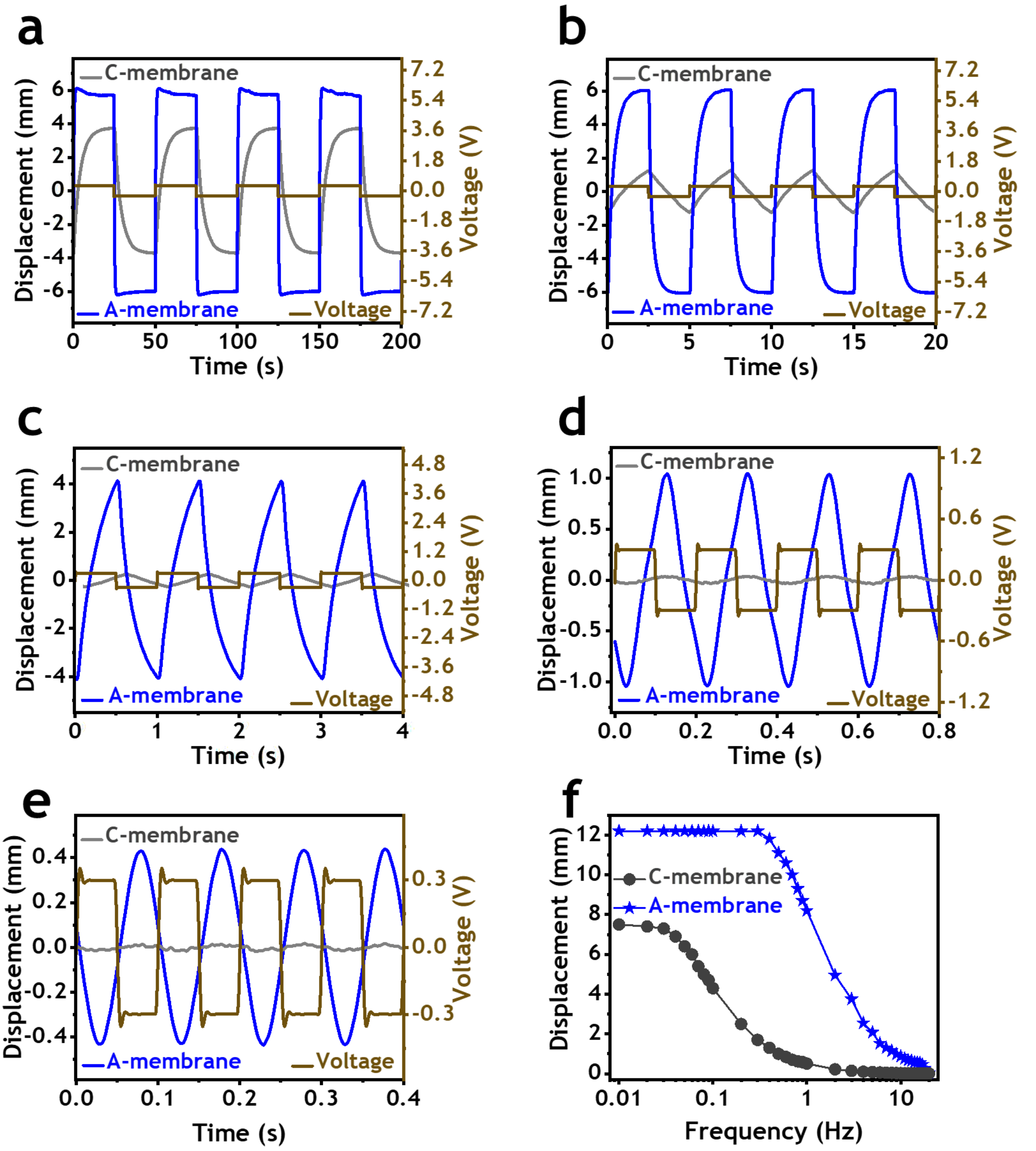

Supplementary Fig. 14. Bending displacement according to frequency. a. 0.02 Hz. **b.** 0.20 Hz. **c.** 1.00 Hz. **d.** 5.00 Hz. **e.** 10.00 Hz. **f.** From 0.01 to 20.00 Hz.

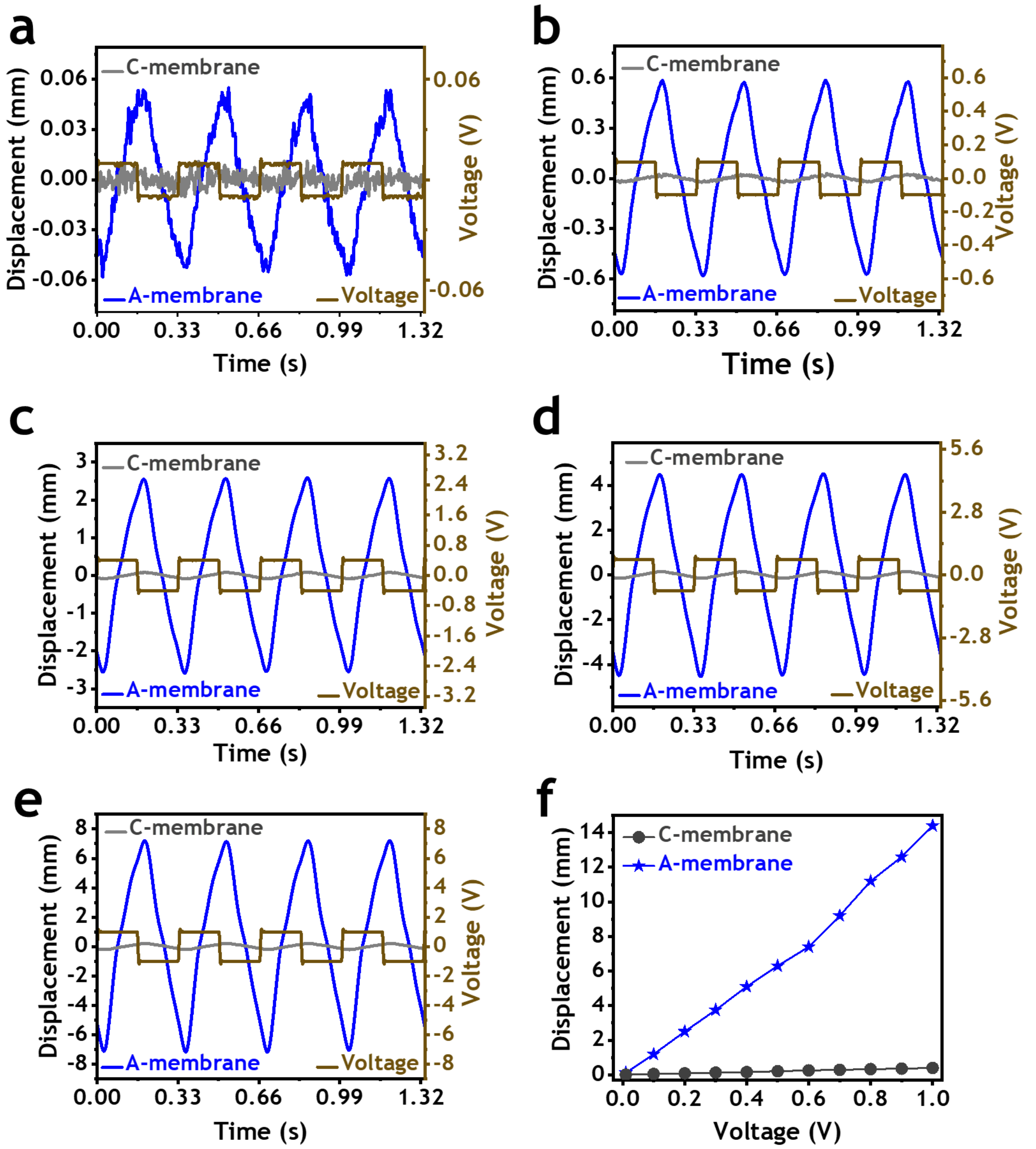

Supplementary Fig. 15. Bending displacement according to voltage. a. 0.01 V. **b.** 0.10 V. **c.** 0.40 V. **d.** 0.70 V. **e.** 1.00 V. **f.** From 0.01 to 1.00 V.

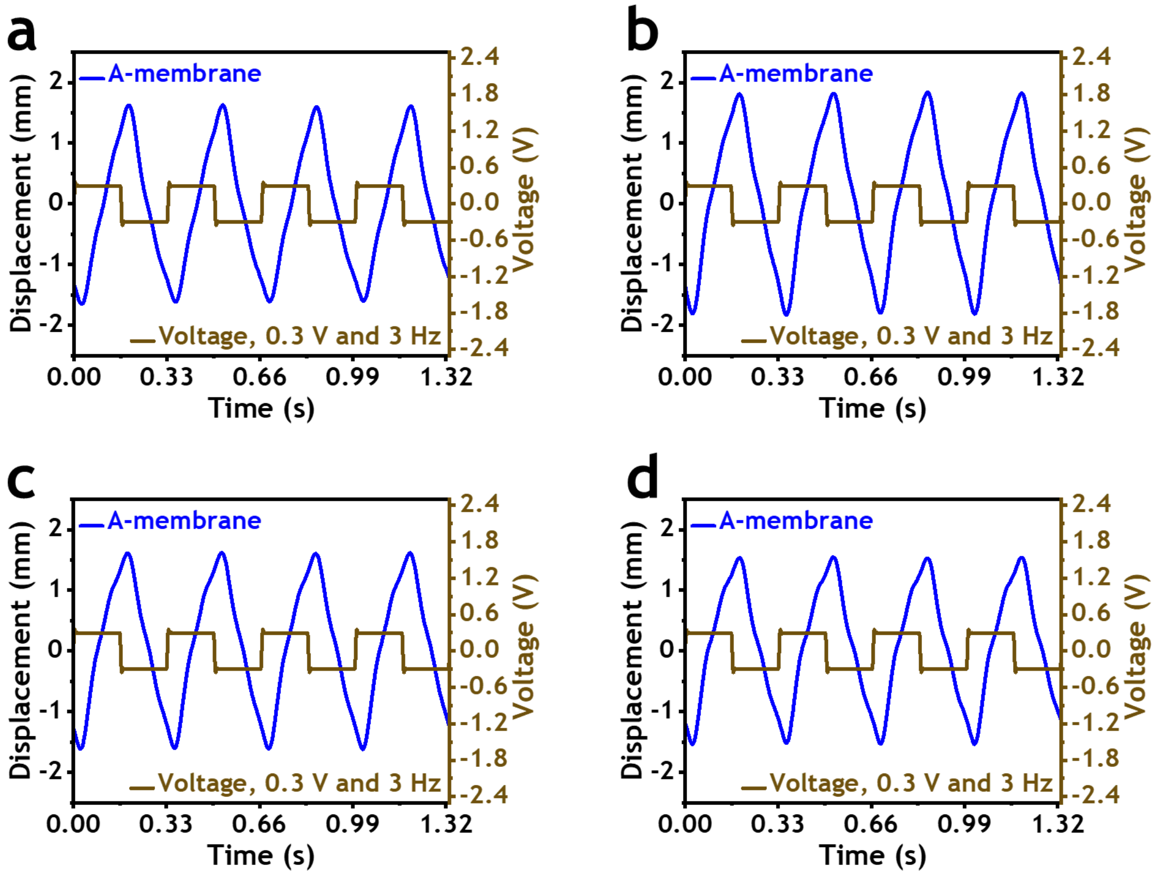

Supplementary Fig. 16. Durability A-membrane actuator at 0.3 V and 3.0 Hz. After **a.** 2 days. **b.** 10 days. **c.** 20 days. **d.** 40 days.

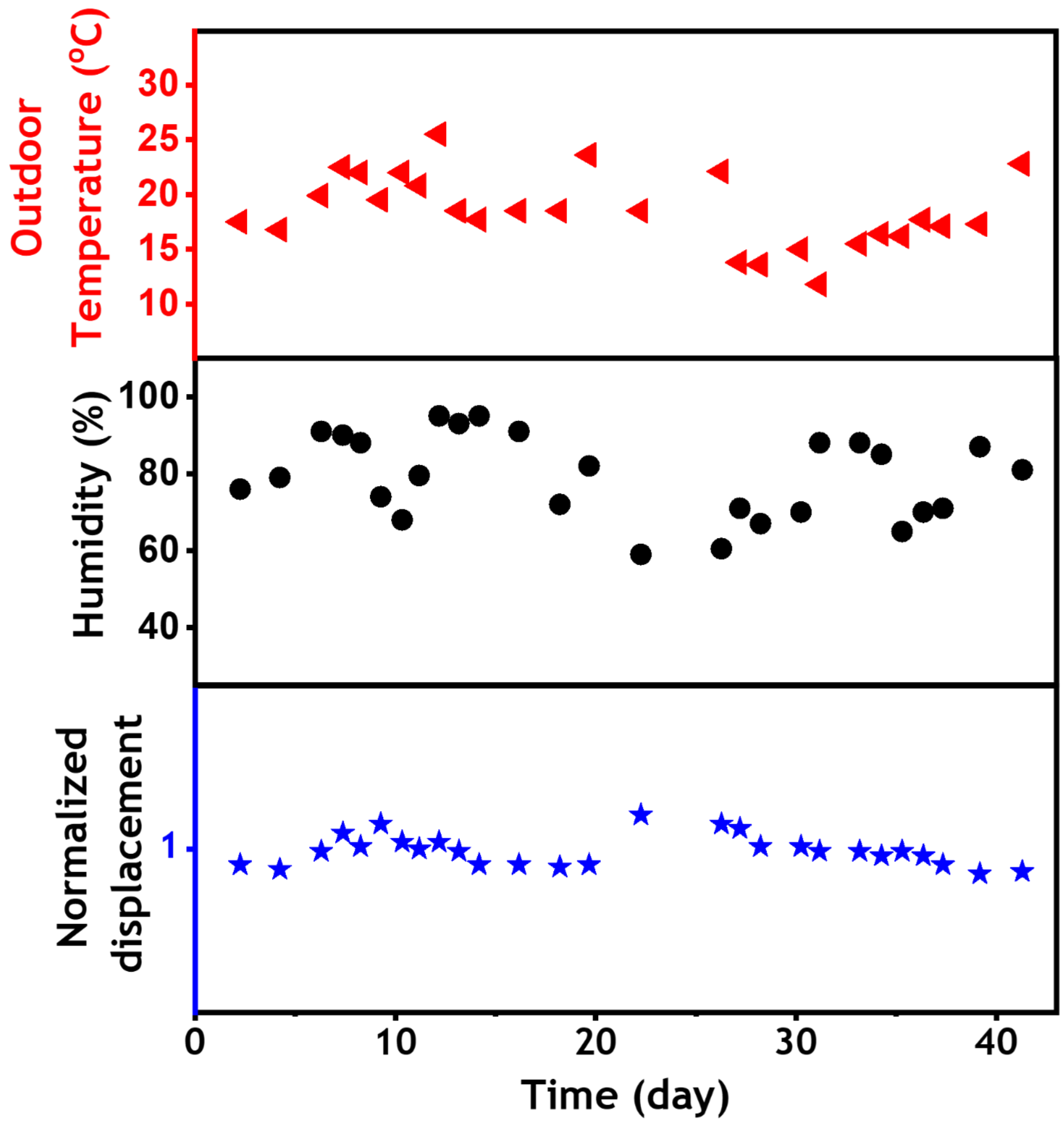

Supplementary Fig. 17. Durability of A-membrane actuator, humidity, and outdoor temperature. The temperature in the testing room was maintained between 18 and 20 °C. The humidity was between 60 and 90 %.

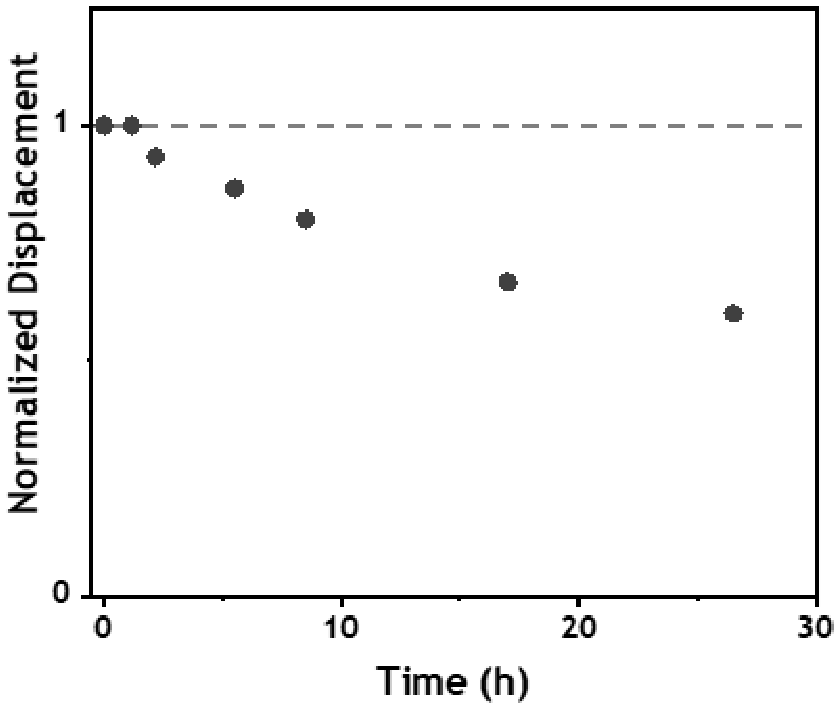

Supplementary Fig. 18. Durability of C-membrane actuator at 1.5 V and 3.0 Hz, normalized to the initial bending displacement.

Supplementary Table 1. Solvent systems for preparing Nafion dispersions.

Solvent systems	Solvents [g]				Nafion [g]
	water	1-propanol	DMAc	$EMImBF_4$	
**A-membrane**	18.00	0.32	1.50	0.22	0.14
**C- membrane**	0.00	0.00	20.00	0.12	0.20

Supplementary Table 2. Hansen solubility parameters and distance parameters of Nafion and solvents.

Materials	Hansen solubility parameters [$MPa^{1/2}$]				Distance parameter, R_a [$MPa^{1/2}$]
	δ_t	δ_d	δ_p	δ_h	
Nafion[1-3]	23.5	17.4	12.5	9.6	$2.69 < R_0 < 20.09$
A-membrane[a)]	45.02	15.58	15.39	38.95	29.72
C-membrane	22.82	16.81	11.52	10.21	1.66
Water[2]	47.8	15.6	16.0	42.3	33.08
1-propanol[2]	24.5	16.0	6.8	17.4	10.06
2-propanol[2]	23.5	15.8	6.1	16.4	9.87
$EMImBF_4$[4]	26.2	17.9	14.8	12.2	3.61
DMAc[2]	22.8	16.8	11.5	10.2	1.67
NMP[2,3]	22.9	18.0	12.3	7.2	2.69
Water/2-propanol[2,3]	35.7	15.7	11.1	29.6	20.09

[a)]this study

Supplementary Table 3. Rise time and durability of the developed actuator and in the literature.

Electro-active ionic actuators			Durability (cycle, frequency) [day]	Rise time (voltage) [s]
Electrolytes	Electrodes	Thickness [μm]		
Nafion/$EMImBF_4$[a)]	PEDOT:PSS	100	42 (>10^7, 3 Hz)	0.9 (0.3 V)
0.5M H_2SO_4 solution[5]	MoS_2/Kapton	26	3.0 (8000, 0.03 Hz)	200 (0.6 V)
COF-DT-SO_3Na[6]	PEDOT:PSS	60	2.72 (23490, 0.1 Hz)	1 (0.5 V)
g-CN/PVDF/$EMImBF_4$[7]	g-CN	85	1.15 (100000, 1 Hz)	200 (3.0 V)
PMMA gel/$LiClO_4$[8]	polypyrrole	220	0.46 (120000, 3 Hz)	>10 (1.0 V)
Nafion/$EMImBF_4$[9]	Ni-MOF/PEDOT:PSS	130	0.35 (30000, 1 Hz)	1.5 (1.0 V)
PSS-b-PMB/$HMImPF_6$[10]	CNT	70	0.31 (13490, 0.5 Hz)	80 (3.0 V)
PVDF-co-HFP porous/$EMImBF_4$[11]	PEDOT:PSS	140	0.23 (20000, 1 Hz)	2.5 (0.5 V)
Nafion/$EMImBF_4$[12]	NSCOF/PEDOT:PSS	110	0.23 (20000, 1 Hz)	2.3 (0.5 V)
Nafion/$EMImBF_4$[13]	Triazine-PIM-1 CTF/ PEDOT:PSS	80-115	0.17 (15000, 1 Hz)	10 (0.5 V)
PS-b-PSS-EMIm/$EMImBF_4$[14]	N,S co-doped graphene/PEDOT:PSS	100	0.16 (14000, 1 Hz)	5 (0.5 V)
Nafion/$EMImBF_4$[15]	MXene/ PEDOT:PSS	110	0.21 (18000, 1 Hz)	1 (0.5 V)
	PEDOT:PSS	110	0.15 (12600, 1 Hz)	14 (0.5 V)
1M LiCl or NaCl solution[16]	V_2O_5 nanofiber	11	-	>100 (1.0 V)
1M NaCl solution[17]	CNT/PVC/CNT	245	-	>15 (1.0 V)
**Other actuators**				
**Names**		**Materials**		
Fuel-power artificial muscles[18]		NiTi SMA/Pt<br>CNT/Pt/Nafion	-<br>-	45<br>1800
Polymer composite humid actuator[19]		Polypyrrole	-	300
Photomechanical actuator[20]		CNT/PDMS	-	10
Liquid crystal photoactuator[21]		Liquid crystal polymer	-	2400
CNT/thermalplastic actuator[22]		CNT/Morthane	-	5
Liquid-crystal elastomer (LCE) thermal actuator[23]		LCE	-	360
Liquid-crystal elastomer (LCE) photo actuator[24]		LCE	-	1.3
Dielectric elastomer, applied voltage 4 - 6 kV[25]		Acrylic, silicone	-	0.001
Shape memory alloys (SMA) actuator[26]		SMA	-	5
CNT yarn muscles[27]		CNT	1.54 (>$2x10^6$, 15 Hz)	-
Fishing line and sewing thread muscles[28]		CNT/nylon	13.89 (>$1.2x10^6$, 1 Hz)	-

[a)]this study

Supplementary References

1 Rynkowska, E. *et al.* Effect of the polar-nonpolar liquid mixtures on pervaporative behavior of perfluorinated sulfonic membranes in lithium form. *J. Membr. Sci.* **518**, 313-327 (2016).

2 Hansen, C. M. *Hansen Solubility Parameters*. (CRC press Taylor & Francis Group, 2007).

3 Welch, C. *et al.* Nafion in dilute solvent systems: dispersion or solution? *ACS Macro. Lett.* **1**, 1403-1407 (2012).

4 Weerachanchai, P., Wong, Y., Lim, K. H., Tan, T. T. & Lee, J. M. Determination of solubility parameters of ionic liquids and ionic liquid/solvent mixtures from intrinsic viscosity. *ChemPhysChem* **15**, 3580-3591 (2014).

5 Acerce, M., Akdogan, E. K. & Chhowalla, M. Metallic molybdenum disulfide nanosheet-based electrochemical actuators. *Nature* **549**, 370-373 (2017).

6 Yu, F. *et al.* Ionic covalent organic framework based electrolyte for fast-response ultra-low voltage electrochemical actuators. *Nat. Commun.* **13**, 390 (2022).

7 Wu, G. *et al.* Graphitic carbon nitride nanosheet electrode-based high-performance ionic actuator. *Nat. Commun.* **6**, 7258 (2015).

8 Madden, J. D., Cush, R. A., Kanigan, T. S. & Hunter, I. W. Fast contracting polypyrrole actuators. *Synth. Met.* **113**, 185-192 (2000).

9 Mahato, M. *et al.* A Dual-responsive magnetoactive and electro-ionic soft actuator derived from a nickel-based metal-organic framework. *Adv. Mater.* **34**, e2203613 (2022).

10 Kim, O., Shin, T. J. & Park, M. J. Fast low-voltage electroactive actuators using nanostructured polymer electrolytes. *Nat. Commun.* **4**, 2208 (2013).

11 Raza, U. *et al.* Micro-structured porous electrolytes for highly responsive ionic soft actuators. *Sens. Actuators B Chem.* **352**, 131006 (2022).

12 Mahato, M. *et al.* Sulfur- and nitrogen-rich porous pi-conjugated COFs as stable electrode materials for electro-ionic soft actuators. *Adv. Funct. Mater.* **30**, 2003863 (2020).

13 Mahato, M. *et al.* CTF-based soft touch actuator for playing electronic piano. *Nat. Commun.* **11**, 5358 (2020).

14 Nguyen, V. H. *et al.* Electroactive artificial muscles based on functionally antagonistic core-shell polymer electrolyte derived from PS-b-PSS block copolymer. *Adv. Sci. (Weinh)* **6**, 1801196 (2019).

15 Umrao, S. *et al.* MXene artificial muscles based on ionically cross-linked Ti3C2Tx electrode for kinetic soft robotics. *Sci. Robot.* **4**, eaaw7797 (2019).

16 Gu, G. *et al.* V2O5 nanofibre sheet actuators. *Nat. Mater.* **2**, 316-319 (2003).

17 Baughman, R. H. *et al.* Carbon nanotube actuators. *Science* **284**, 1340-1344 (1999).

18 Ebron, V. H. *et al.* Fuel-powered artificial muscles. *Science* **311**, 1580-1583 (2006).

19 Ma, M., Guo, L., Anderson, D. G. & Langer, R. Bio-inspired polymer composite actuator and generator driven by water gradients. *Science* **339**, 186-189 (2013).

20 Ahir, S. V. & Terentjev, E. M. Photomechanical actuation in polymer-nanotube composites. *Nat. Mater.* **4**, 491-495 (2005).

21 Iamsaard, S. *et al.* Conversion of light into macroscopic helical motion. *Nat. Chem.* **6**, 229-235 (2014).

22 Koerner, H., Price, G., Pearce, N. A., Alexander, M. & Vaia, R. A. Remotely actuated polymer nanocomposites--stress-recovery of carbon-nanotube-filled thermoplastic elastomers. *Nat. Mater.* **3**, 115-120 (2004).

23 Pei, Z. *et al.* Mouldable liquid-crystalline elastomer actuators with exchangeable covalent bonds. *Nat. Mater.* **13**, 36-41 (2014).

24 Camacho-Lopez, M., Finkelmann, H., Palffy-Muhoray, P. & Shelley, M. Fast liquid-crystal elastomer swims into the dark. *Nat. Mater.* **3**, 307-310 (2004).

25 Pelrine, R., Kornbluh, R., Pei, Q. & Joseph, J. High-speed electrically actuated elastomers with strain greater than 100%. *Science* **287**, 836-839 (2000).

26 Oh, S. *et al.* Cooling-accelerated nanowire-nitinol hybrid muscle for versatile prosthetic hand and biomimetic retractable c arrhenius. *Adv. Funct. Mater.* **32,** 2111145 (2021).

27 Lima, M. D. *et al.* Electrically, chemically, and photonically powered torsional and tensile actuation of hybrid carbon nanotube yarn muscles. *Science* **338**, 928-932 (2012).

28 Haines, C. S. *et al.* Artificial muscles from fishing line and sewing thread. *Science* **343**, 868-872 (2014).
