# Supplementary material for: Functionally antagonistic polyelectrolyte for electro-ionic soft actuator
Source: Nat Commun. 2024 Jan 10;15:435. doi: 10.1038/s41467-024-44719-z (PMC10781978; doi:10.1038/s41467-024-44719-z)
Supplement: Supplementary file 3 — Description of Additional Supplementary Files [file 41467_2024_44719_MOESM3_ESM.pdf]

Description of Additional Supplementary Files

Supplementary Movie 1: Three-dimensional Nafion micelle in water by reconstructing from raw images of cryo-TEM tomography.

Supplementary Movie 2: Three-dimensional Nafion micelle in water by reconstructing from cryo-TEM tomography using software.

Supplementary Movie 3: Bending displacement at 1.0 V and 0.1 Hz.

Supplementary Movie 4: Crawling inchworm-mimetic soft robot speeded up by six times.

Supplementary Movie 5: Tensegrity component moved by electro-ionic actuators.
